# Fatty acid synthase in chemoresistance: mechanisms and therapeutic opportunities

**DOI:** 10.3389/fphar.2025.1674752

**Published:** 2025-09-11

**Authors:** Li Huang, Mingjuan Zhang, Yadong Xiao

**Affiliations:** Guangzhou Vocational University of Science and Technology, Guangzhou, Guangdong, China

**Keywords:** chemoresistance, lipid metabolism, fatty acid synthase, drug discovery, mechanisms and therapeutic opportunities

## Abstract

Chemoresistance has been a major obstacle to the efficient treatment of cancer. Recently, targeting lipid metabolism has gained significant attention because of its roles not only in promoting cancer progression but also in inducing chemotherapy resistance. Fatty acid synthase (FAS) is the sole enzyme that is in charge of catalyzing the synthesis of palmitate, a long-chain lipid that is essential for membrane construction and post-translational modification in cell biology. Both FAS and its product, palmitate, have been validated as critical players in mediating or causing chemoresistance in cancers, although the details remain elusive, requiring further basic studies. In this mini-review, we provide a brief and concise overview of the basic research on FAS in cancer and its mechanisms of inducing chemoresistance. More importantly, we summarize and critically discuss the progress of small-molecule FAS inhibitors, especially those in clinical trials. While by far, several FAS inhibitors, including denifanstat and omeprazole, have demonstrated beneficial effects in clinical trials, no candidate has been approved by the FDA. We concluded here that targeting FAS is a feasible strategy to overcome chemoresistance, although more interdisciplinary efforts are needed to identify a potent, specific, and bioavailable FAS inhibitor for clinical applications.

## 1 Introduction

Fatty acid synthase (FAS) is a multifunctional enzyme complex responsible for the *de novo* synthesis of long-chain saturated fatty acids, primarily palmitate, using acetyl-CoA and malonyl-CoA (Fhu and Ali, 2020). FAS is composed of several catalytic domains that work sequentially to synthesize palmitate, including β-ketoacyl synthase (KS), malonyl/acetyl transferase (MAT), dehydratase (DH), enoyl reductase (ER), β-ketoacyl reductase (KR), acyl carrier protein (ACP), and thioesterase (TE) (Herbst et al., 2018). Under normal physiological conditions, FAS expression is relatively low in most adult tissues due to dietary fat intake that can meet metabolic needs. However, in many types of cancers, including breast, prostate, lung, colorectal, and ovarian cancers, FAS is dramatically upregulated not only to support the rapid progression but also closely involved with aggressive tumor phenotypes and correlated with poor clinical outcomes (Menendez and Lupu, 2007; Esslimani-Sahla et al., 2007). This metabolic reprogramming is a hallmark of cancer, which is characterized by the ability to support proliferative signaling and resist cell death by reprogramming energy and biosynthetic pathways (Yang et al., 2023; Zhou et al., 2024; Vanauberg et al., 2023).

One of the primary functions of FAS in cancer cells is to produce palmitate, which subsequently serves as a key building block for membrane phospholipids and lipid-modified proteins (Liu et al., 2008). Rapidly proliferating tumor cells require constant synthesis of cellular membranes to support continuous and unleashed cell division, proliferation, and migration. The palmitate produced by FAS provides essential lipid components for membrane biogenesis, which in turn facilitates mitotic progression, cell growth, and survival. Additionally, palmitate and other lipids derived from FAS activity contribute to the formation of lipid rafts that play critical roles in cell signaling, trafficking, and intercellular communication (Vanauberg et al., 2023).

Beyond its role in membrane synthesis, FAS also supports energy storage and redox balance in cancer cells. The fatty acids synthesized by FAS can be esterified into triglycerides and stored in lipid droplets, serving as a reservoir of energy-rich molecules that can be mobilized under nutrient-deprived or stress conditions (Jensen-Urstad and Semenkovich, 2012). This lipid storage contributes to the metabolic plasticity of cancer cells, allowing them to survive harsh tumor microenvironments of hypoxia or intensive oxidative stress, etc (Weng et al., 2024). Moreover, the reduction of NADPH during fatty acid synthesis can buffer oxidative stress by maintaining cellular redox homeostasis, which is another key survival mechanism in cancer biology (Weng et al., 2024).

In addition to its roles in palmitate production and energy storage for cancer cells, FAS can also function as a pro-growth and survival factor for cancer cells, promoting the initiation, progression, development, and migration of cancers by integrating other critical signaling pathways. In general, FAS may promote cancers in two ways, including (1) palmitate-mediated modifications of signaling lipids or proteins (Louie et al., 2013), and (2) its direct interactions with other critical signal pathways.

The palmitate generated from FAS may facilitate the construction of certain signaling lipids, including lysophosphatidic acids and ceramide-1-phosphate, both of which closely engage in key signaling pathways that promote cancer initiation and progression (Arana et al., 2010; Xu, 2019; Benjamin et al., 2015). Another major role palmitate play is the protein palmitoylation mediated by palmitoyl CoA, a common but essential posttranslational modification that can help improve the binding affinity, stabilize, and transport certain pro-cancer proteins (Resh, 2013; Zhou et al., 2023). For example, it has been found that the palmitoylation of epidermal growth factor receptor (EGFR) can induce tyrosine kinase inhibitor gefitinib resistance in non-small cell lung cancer (NSCLC) (Ali et al., 2018). The palmitoylation of Hedgehog proteins at the N-terminal, influences membrane binding and regulates the signaling range and efficacy (Buglino and Resh, 2012). In addition to the two aforementioned pivotal proteins, other key players, such as Wnt and Src, can also undergo palmitoylation to exert their functions in promoting signaling transduction and cancer progression (Buglino and Resh, 2012; Resh, 2017; Resh, 2021). A recent study showed that palmitic acid from the diet could induce epigenetic changes of intratumoural Schwann cells, i.e., Set1A/COMPASS activation (Pascual et al., 2021). It is noteworthy that Set1A/COMPASS activation is triggered by dietary palmitic acid rather than the *de novo* generated by FAS (Pascual et al., 2021), however, we suspect that it may employ the same mechanism, requiring further validation.

Another important role for FAS is that it can directly or indirectly participate in promoting cancer cells’ growth via regulating specific signaling pathways, although the exact acting mode remains elusive. A recent study indicated that FAS is essential in the 2D-to-3D growth transition of breast cancer cells, via an isocitrate dehydrogenase 1 (IDH1)- and reactive oxygen species (ROS)-dependent pathways (Bueno and Quintela-Fandino, 2020). In colorectal cancer, FAS was found to enhance cancer cells’ proliferation and lymph node metastasis, causing a poor prognosis (Lu et al., 2019). This cell-based study also found that FAS can increase ATP production via suppressing AMP-activated protein kinase (AMPK)/mechanistic target of rapamycin (mTOR) signals (Lu et al., 2019). Similar mechanisms were also verified in gastric and ovarian cancers (Sun et al., 2018; Wagner et al., 2017). Furthermore, FAS overexpression appears to coordinate with *PTEN*, a tumor-suppressing gene, to cause an aggressive phenotype in murine prostate and prostate cancer patients, although more details are largely unknown (Bastos et al., 2021). In addition, FAS is able to regulate epithelial-mesenchymal transition (EMT) of breast cancer cells via liver fatty acid-binding protein (L-FABP) and VEGF/VEGFR-2 mediated mechanism (Li et al., 2014a).

However, it should be noted that although current evidence supports multiple and critical roles of FAS in regulating cancer cell growth and survival, potentially through modulation of key signaling pathways, its precise molecular modes of action remain unclear. Similarly, the mechanistic basis of FAS overexpression in coordination with *PTEN* loss is largely unknown. Addressing these gaps will require in-depth studies integrating pathway dissection, protein–protein interaction mapping, and advanced lipidomic analyses.

## 2 FAS confers chemotherapy resistance in cancers

The development of multidrug resistance (MDR) significantly limits the efficacy of literally all anticancer treatments and impacts patients’ survival and quality of life, despite the significant progress of available and cutting-edge treatments (Wang et al., 2021b; Dong et al., 2022; Zhao et al., 2024). Drug resistance accounts for approximately 90% of deaths among cancer patients, suggesting an urgent and unmet clinical need that requires swift action and interdisciplinary effort to tackle via developing novel therapeutic agents (Cui et al., 2022; Yan et al., 2024). Previously, it was known that FAS induces drug resistance via its major role in palmitate production that supports the survival and proliferation of cancer cells (Liu et al., 2008). Over the past decade, more details have revealed the involvement of other functions.

FAS overexpression can cause drug and radiation resistance via activating DNA repair through the nonhomologous end-joining (NHEJ) pathway and increasing the expression and activity of poly(ADP-ribose) polymerase 1 (PARP-1) by the inhibition of NF-κB and the enhancement of the transcription factor specificity protein 1 (Sp1) (Wu et al., 2016). FAS was also found to reverse apoptosis effects and ceramide over-production, both of which were induced by doxorubicin, through the inactivation of caspase 8 and neutral sphingomyelinase that was directly involved in cellular ceramide synthesis (Liu et al., 2013). In addition, FAS seems to be able to induce MDR in cancers (Wu et al., 2016; Liu et al., 2013), especially those DNA-damaging drugs but not microtubule modulators (Liu et al., 2013). These facts may suggest a common mechanism, i.e., DNA-damage response such as the upregulation of PARP-1 due to FAS, which has therapeutic implications to develop novel combination strategy to treat resistant cancers (Wu et al., 2014).

A recent study showed that the overexpressed FAS induced anoikis resistance in gastric cancer via the p-ERK1/2/Bcl-xL pathway, whereas the silencing of FAS reversed anoikis resistance and retarded the migration and invasion of gastric cancer cells (Yu et al., 2021). In tyrosine kinase inhibitor gefitinib-resistant NSCLC PC-9GR cells that possesses EGFR delE746-A750 mutation, mRNA levels and protein expressions of both FAS and its regulator sterol regulatory element-binding transcription factor 1 (SREBF1) were found to be significantly elevated (Ali et al., 2018). The silencing of EGFR could reverse the enhanced FAS and SREBF1, suggesting a network of FAS and EGFR, which was then validated by further study which showed FAS facilitated the palmitoylation of EGFR, leading to gefitinib resistance (Ali et al., 2018). In breast cancer cell line MCF-7-MEK5 with stably EMT property, FAS was able to regulate the sensitivity of tumor necrosis factor-α (TNF-α) through modulating TNF receptor 2 (TNFR2) via lipid rafts and activating Wnt-1/β-catenin signaling pathway that closely involves in EMT (Li et al., 2014b).

## 3 FAS as a feasible target validated by genomic knockdown or knockout

FAS is universally upregulated to meet the needs of cancer cells, regardless of types, and combat cell death induced by chemotherapy, leading to drug resistance. Meanwhile, growing evidence has suggested that the downregulation of FAS, by short hairpin RNA (shRNA) or small interfering RNA (siRNA), can suppress cancer phenotype and, importantly, reverse drug resistance in cancers, providing direct evidence supporting FAS as a feasible target for cancers. We here listed a few of these studies in different cancer types.

In breast cancer patients with invasive ductal carcinoma, the level of FAS correlated with metastasis and invasion, while the silence of FAS in SK-Br-3 cells led to decreased fatty acids and decreased migration (Xu et al., 2021). Decreased cell viability was also discovered in breast cancer MCF-7 cells transfected with FAS siRNA (Pham et al., 2021), and it also sensitized doxorubicin, accompanied by decreased PARP1 (Wu et al., 2014). FAS expression status correlates with the malignant phenotype during breast cancer progression. FAS shRNA treatment reduces the secretion of vascular endothelial growth factor (VEGF) and angiogenesis in breast cancer CA1d cells, leading to reduced tumor growth and a dormant-like phenotype *in vivo* (Gonzalez-Guerrico et al., 2016). FAS was upregulated in mantle cell lymphoma Jeko-1, Mino, SP53 and Rec-1, and its downregulation by FAS inhibitor or siRNA suppressed cell growth (Gelebart et al., 2012). In addition, FAS knockout simultaneously downregulate β-catenin whose over-expression may simultaneously increase FAS, suggesting an interaction between these two and/or a combination strategy (Gelebart et al., 2012). Similarly, in liver cancer HepG2 cells, FAS siRNA treatment leads to decreased cells proliferation and increased apoptosis mediated by downregulated Bcl-2, upregulated Bax, caspase-3, and P21, which was also accompanied with decreased β-catenin and c-Myc (Zhang et al., 2020). In NSCLC A549 cells, FAS siRNA transfection could reduce the proliferation rate, migration and invasion ability through reducing ATP and lactic acid productions, suggesting a connection between fatty acid and glucose metabolism (Chang et al., 2019). More importantly, FAS siRNA treatment could inhibit tumor growth in the A549 cells xenograft mouse model (Chang et al., 2019). FAS upregulation could stimulate the proliferation and metastasis of colorectal cancer SW480 and HCT116 cells, while the knockdown of FAS by shRNA reduced the proliferation and migration (Lu et al., 2019). Similarly, in colorectal cancer KM20 and HT29 cells, FAS shRNA was able to suppress the proliferation and colony numbers via attenuating CD-44/c-Met signal (Zaytseva et al., 2012). Furthermore, the silence of FAS could reduce the tumor growth and inhibit the metastasis in mice xenograft models (Zaytseva et al., 2012). FAS also play key role in retinoblastoma Y79, WERI RB1 cells, and the silence of FAS by siRNA lead to decreased cell viability and increased apoptosis (Sangeetha et al., 2015). This study also indicated a close interaction between FAS and the phosphoinositide 3-kinase (PI3K)/protein kinase B (AKT), indicating a therapeutic implication via dual-targeting. In uterine leiomyosarcomas SK-UT-1 cells, the upregulation of FAS appears to activate the histone methylation of H3K9 (H3K9me3) and acetylation of H3K27 (H3K27ac), therefore promoting the proliferation, which can be reversed by FAS siRNA transfection (Guan et al., 2017). Furthermore, gastric cancer SGC-7901 transfected with FAS siRNA demonstrated a slower proliferation rate and migration ability than the non-treated cells (Sun et al., 2018). FAS siRNA treatment negatively impacted the proliferation and the migration of bladder carcinoma UMUC3 cells (Yan et al., 2019), 5637 and 253J cells (Zheng et al., 2016) via inducing apoptosis (Jiang et al., 2012).

The knockdown of FAS could also modulate the sensitivity of certain chemotherapeutics. In TNBC MDA-MB-231 cells that show cisplatin-resistant property, FAS siRNA treatment increased cisplatin sensitivity (Al-Bahlani et al., 2017). Recently, a novel liposome composed with FAS siRNA and a HER2-targeting fab’ fragment was shown to selectively target HER2^+^ SK-BR3 and MCF-7 cells, with decreased proliferation and migration (Khan et al., 2020). FAS levels in melanoma LM16 R cells were found to negatively correlate with IC_50_ values of Vemurafenib (an approved BRAF inhibitor), and the silence of FAS could enhance the cytotoxicity of PLX4032 to PLX4032-resistant LM16 R cells (Stamatakos et al., 2021).

The information above taken together strongly suggests that FAS is a pharmacological target for cancers, including chemo-resistant cancers.

## 4 FAS-targeting drug candidates for overcoming chemoresistance in cancers

**FIGURE 1:**
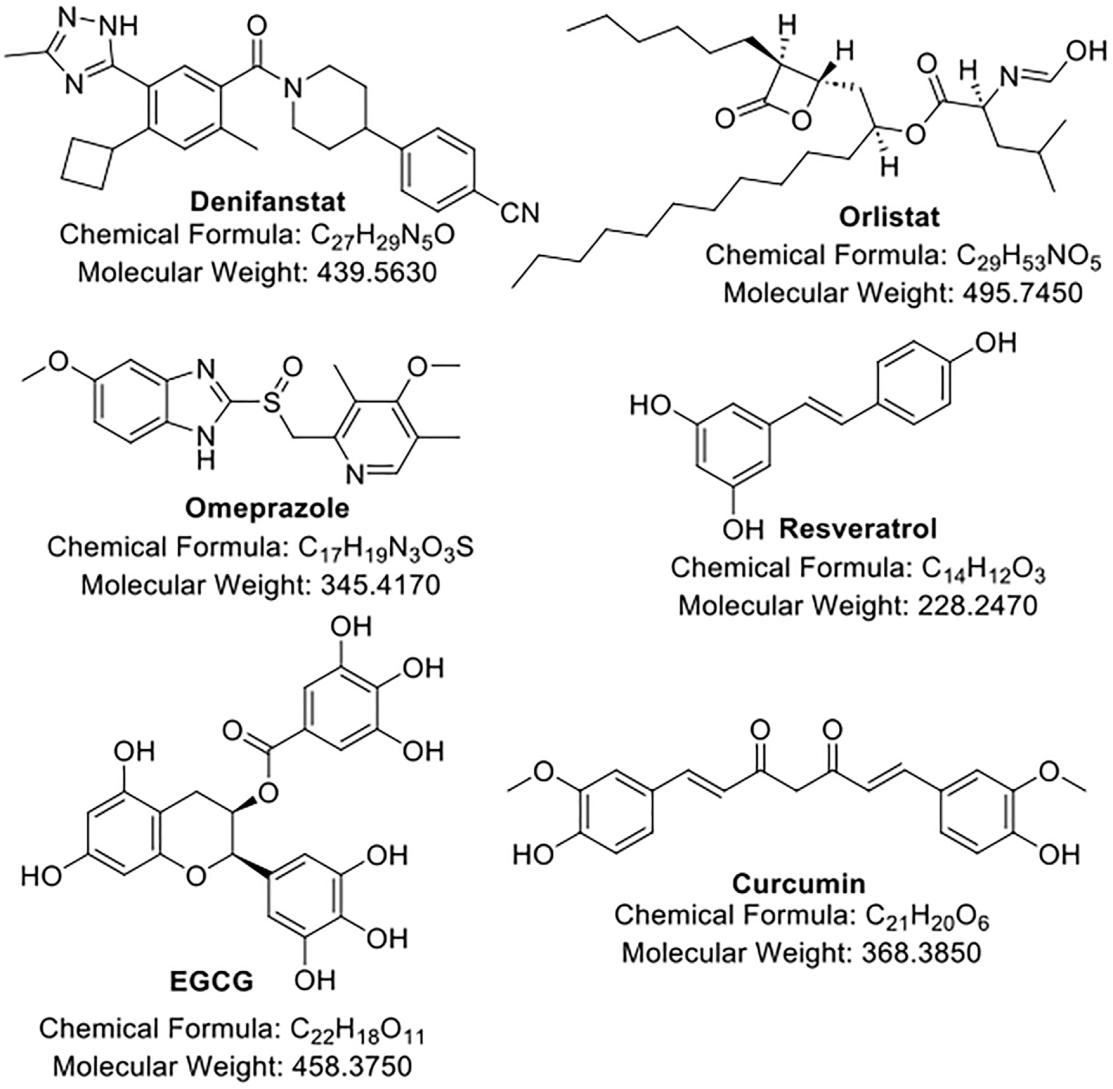
The structures of FAS inhibitors.

In this section, we summarize and discuss the recent progress of the development of drug candidates (Figure 1; Table 1) that are either prominent or active in clinical trials for their application in overcoming chemoresistance in cancers. We also discussed the challenges each candidate faced.

**TABLE 1:** FAS inhibitors in combinations show promising therapeutic effects in clinical trials.

Candidates	Combinations	Clinical status/effects	References
Denifanstat	Bevacizumab	Well-tolerated, favorable safety profile and response signals when combined with bevacizumab	Kelly et al. (2023)
	Paclitaxel	Manageable toxicity and preliminary antitumor activity	Falchook et al. (2021)
	Enzalutamide	Ongoing	Beer et al. (2017)
Orlistat	NA	NA	NA
Omeprazole	Anthracycline/taxane	Promising pathologic complete response	Sardesai et al. (2021)
	Docetaxel/cisplatin	Enhanced antitumor effects	Wang et al. (2015)
EGCG	NA	NA	NA
Curcumin	Docetaxel	Encouraging efficacy results	Bayet-Robert et al. (2010)
Resveratrol	NA	NA	NA

Note: NA, not available.

Denifanstat, previously known as TVB-2640 or ASC40, is an investigational, oral inhibitor of the FAS KR domain developed by Sagimet Biosciences. Different with the other inhibitors discussed below, denifanstat appears to be an optimized compound and is the only one that has undergone extensive medicinal chemistry study (a critical step in developing a new therapeutic agent), and it is possible third-generation of this class FAS inhibitor according to the disclosed patent (PCT/GB2007/004920, titled “Sulfonamide derivatives for therapeutic use as fatty acid synthase inhibitors”).

Denifanstat shows potency in impairing membrane synthesis, energy homeostasis, and oncogenic lipid signaling mediated by FAS inhibition, making it a compelling candidate. In TNBC brain metastases, combining denifanstat with topoisomerase inhibitor SN-38 suppressed tumor progression more effectively than either agent alone by simultaneously targeting angiogenesis and metabolic reprogramming (Serhan et al., 2024). In clinical settings, denifanstat has shown encouraging results, especially in combination regimens. A first-in-human Phase 1 study (NCT02223247) evaluated denifanstat as monotherapy and in combination with paclitaxel in patients with advanced solid tumors, revealing manageable toxicity and preliminary antitumor activity, with notable responses in patients harboring KRAS mutations (Falchook et al., 2021). Building on this, a Phase 2 trial (NCT03808558) is ongoing to investigate the single use of denifanstat in KRAS-mutant NSCLC (Salgia et al., 2021). In another Phase 2 study (NCT03032484), denifanstat was administered with bevacizumab targeting vascular endothelial growth factor A (VEGF-A) in patients with recurrent high-grade astrocytomas, including glioblastoma, targeting both metabolic and angiogenic pathways, and the results showed that the combination was safe and promising signals were detected in treated patients, warranting further study (Kelly et al., 2023). Additionally, a Phase 1b trial (NCT05743621) is evaluating denifanstat with enzalutamide (an androgen receptor inhibitor) in metastatic castration-resistant prostate cancer (mCRPC) (Beer et al., 2017); however, no further details of its outcomes have been revealed. Denifanstat represents the first orally active and specific FAS inhibitor, although very few preclinical results have been published. Further basic and preclinical studies as well as its combinations with other anticancer agents should be largely exploited for its role and applications in reversing chemoresistance, including the cutting-edge immunotherapy.

Orlistat, a well-known anti-obesity drug approved by the FDA and reproposed as a FAS TE domain inhibitor, has been studied in multiple preclinical cancer studies to enhance chemotherapy efficacy. In prostate cancer models, Orlistat combined with docetaxel led to synergistic inhibition of tumor cell proliferation and increased apoptosis by blocking lipid biosynthesis, promoting proapoptotic caspase activation, and enhancing microtubule stabilization, which was also independent of ABCB1 (Souchek et al., 2023; Souchek et al., 2017). In colon cancer, co-treatment with oxaliplatin and Orlistat significantly reduced tumor growth and induced autophagy through cell cycle arrest and increased apoptosis, indicating a metabolic vulnerability exploited by dual therapy (Zhang et al., 2022). In pancreatic cancer cells, combining Orlistat with gemcitabine induced endoplasmic reticulum stress, increased DNA damage, and reduced cell viability by interfering with fatty acid metabolism and nucleotide biosynthesis (Tadros et al., 2017). However, currently, no active clinical trials using orlistat are scheduled for cancers. We suspected that it may not be potent enough to achieve favorable treatment outcomes since it is originally a drug to treat obesity. Plus, orlistat is not a specific FAS inhibitor but rather a lipase inhibitor that reduces the overall absorption of fatty acids. Another issue for orlistat is the low bioavailability due to high lipophilicity. Therefore, further structural modifications are needed to address these issues. Despite the challenges, since orlistat is an approved drug, determining its impact on treatment outcomes in cancer patients is still meaningful.

Several preclinical studies in the past decade have identified proton pump inhibitors (PPIs), especially lansoprazole and omeprazole, as potential FAS TE domain inhibitors (Wang et al., 2021a; Fako et al., 2015). These studies demonstrated that PPIs can bind to and inhibit the TE domain of FAS, leading to disrupted palmitate synthesis, impaired lipid homeostasis, and induction of apoptosis via disturbing DNA repair pathways in cancer cells (Wang et al., 2021a; Fako et al., 2015). In breast, prostate, and colon cancer models, PPI treatment reduced FAS activity, induced DNA damage, and suppressed tumor cell proliferation (Beebe et al., 2022; Fako et al., 2015). A Phase 1/2 clinical trial (NCT01069081) demonstrated that high-dose PPIs, including omeprazole, improved outcomes in metastatic breast cancer patients when added to chemotherapy, by reducing tumor acidity and enhancing drug uptake (Wang et al., 2015). In a Phase 2 trial (NCT02595372) of operable TNBC, omeprazole was safely administered before and during neoadjuvant chemotherapy, leading to a high pathologic complete response (pCR) rate of 72.4% in FAS-overexpressing patients (Sardesai et al., 2021). Omeprazole significantly reduced FAS expression and activity, with no severe toxicities, supporting its potential as a metabolic sensitizer in TNBC treatment (Sardesai et al., 2021). Large-scale clinical trials in certain FAS-dependent, -addicted, or -overexpressed cancer patients, including pretreated patients, are needed to proceed with its final approval as a drug targeting FAS. Similar to orlistat, omeprazole is not a specific FAS inhibitor, given that it is originally developed to target and inhibit the H^+^/K^+^-ATPase enzyme, rendering it also a multitargeting agent. Therefore, its off-target effect should be closely monitored during clinical trials.

Several prominent natural products have also shown FAS-targeting or -downregulating effects. However, they are all multitargeting compounds with multiple potential targets. Since they can thus serve as tool compounds to study pharmacological effects, or as hit compounds that can be further structurally modified to improve specificity and selectivity, they are briefly discussed below.

Epigallocatechin gallate (EGCG) is the most abundant and biologically active catechin in green tea, widely recognized for its antioxidant, anti-inflammatory, and anticancer properties. EGCG has been shown to directly inhibit FAS, particularly in cancer cells where FAS is often overexpressed (Wang and Tian, 2001; Puig et al., 2008; Relat et al., 2012). EGCG has advanced into early-phase clinical trials primarily as a chemopreventive or adjuvant therapy. In a Phase 1 trial (NCT00455416) in patients with early-stage breast cancer, oral EGCG was found to be safe and well tolerated, with evidence of FAS downregulation in tumor tissue and modulation of lipid metabolism (Tuli et al., 2023). In prostate cancer patients, EGCG-rich green tea extracts reduced prostate-specific antigen (PSA) levels and markers of oxidative stress in Phase 2 studies, supporting its role in disease stabilization (Johnson et al., 2010). Additionally, in colorectal cancer prevention trials (NCT01360320), EGCG supplementation reduced the recurrence of adenomas and altered serum lipid profiles (Stingl et al., 2011). However, EGCG’s low bioavailability and rapid metabolism remain limitations for achieving systemic anticancer efficacy (Wang et al., 2022), warranting further research into synthetic derivatives, such as those guided by artificial intelligence (AI).

Curcumin is a polyphenolic compound derived from the spice turmeric (*Curcuma longa*), which has long been recognized for its anti-inflammatory, antioxidant, and anticancer properties. Curcumin has been shown to inhibit FAS, thereby suppressing tumor progression, metastasis, and chemoresistance (Zhao et al., 2011; Fan et al., 2014; Fan et al., 2016; Younesian et al., 2017). Curcumin has been evaluated in numerous clinical trials, mainly for its safety, chemopreventive potential, and ability to enhance conventional cancer therapies (Greil et al., 2018). In a Phase 1/2 trial in patients with advanced colorectal cancer (NCT00118989), curcumin was well tolerated up to 3.6 g/day and showed biological activity by modulating cancer-related biomarkers including cyclin D1, COX-2, and possibly FAS (Fuentes et al., 2017). In breast cancer patients (NCT number is not available), curcumin combined with docetaxel showed an improved clinical response and reduced inflammation; however, direct measurement of FAS modulation was not conducted (Bayet-Robert et al., 2010). Additional clinical studies (NCT00745134, NCT02138955) in pancreatic, prostate, and head and neck cancers have shown that curcumin can improve treatment tolerability and reduce tumor-promoting inflammation and oxidative stress (Panknin et al., 2023). However, similar as EGCG, curcumin’s poor bioavailability limits its systemic efficacy (Anand et al., 2007; Lopresti, 2018), which has prompted the development of enhanced formulations, e.g., liposomal curcumin, nanoparticles, and curcumin analogs by conventional medicinal chemistry or AI-driven technology, that may provide improved FAS inhibition *in vivo*.

Resveratrol is a natural polyphenol found in grapes, red wine, peanuts, and several berries, known for its antioxidant, anti-inflammatory, and chemopreventive properties and has been identified as an FAS inhibitor (Pandey et al., 2011; Wang et al., 2019). There are several clinical studies of resveratrol in cancer (Singh et al., 2015; Howells et al., 2011). In a Phase 1 trial (NCT00256334) in patients with colorectal cancer, daily resveratrol (up to 5 g/day) was well tolerated and led to detectable levels in colon tissue (Nguyen et al., 2009). In patients with multiple myeloma, resveratrol-enriched extracts were associated with immune activation and reductions in inflammatory cytokines (Ren et al., 2025; Bhardwaj et al., 2007). Despite promising signals, resveratrol’s rapid metabolism and low oral bioavailability remain barriers to clinical efficacy (Salehi et al., 2018), requiring studying for specific formulations and synthetic derivatives or analogs to enhance delivery and metabolic stability.

While EGCG, Curcumin, and Resveratrol have demonstrated promising *in vitro* activity in targeting FAS and reversing chemoresistance, their clinical translation is majorly hampered by pharmacokinetic limitations. For instance, EGCG’s poor oral bioavailability and rapid metabolism result in subtherapeutic plasma and tumor concentrations, which are insufficient for sustained FAS inhibition, required to resensitize resistant tumor cells. Similarly, Curcumin’s rapid conjugation and systemic elimination hinder its ability to modulate lipid metabolism and apoptosis pathways involved in chemoresistance. Resveratrol’s instability in plasma limits its capacity to inhibit key survival proteins such as survivin, which contribute to drug resistance. Recent formulation strategies, including nanoparticle encapsulation, liposomal delivery, and structural analog development, have shown improved tumor targeting and prolonged systemic exposure, leading to enhanced chemosensitization in preclinical models. These advances suggest that overcoming these bioavailability barriers is essential for the clinical viability of these compounds as FAS-targeted chemoresistance modulators.

## 5 Conclusion

FAS has been validated as a druggable target for cancer treatment due to its essential roles in promoting cancer cell proliferation and mitigating chemotherapy-induced cell death. While several clinical candidates have been investigated in multiple trials, further efforts are needed to develop more specific and bioactive FAS inhibitors.
